# Myocardial infarction revealing a pleuropericardial cyst: a case report

**DOI:** 10.11604/pamj.2021.39.246.29049

**Published:** 2021-08-18

**Authors:** Saîda Amaqdouf, Chaimae Toutai, Noha El Ouafi, Zakaria Bazid

**Affiliations:** 1 Department of Cardiology, Mohammed VI University Hospital of Oujda, Mohammed First University of Oujda, Oujda, Morocco,; 2 Laboratory of Epidemiology, Clinical Research and Public Health, Faculty of Medicine and Pharmacy, Mohammed the First University of Oujda, Oujda, Morocco

**Keywords:** Pericardial cyst, myocardial infarction, echocardiography, case report

## Abstract

Pericardial cysts are a rare entity, accounting for 6-7 percent of all mediastinal masses. They are frequently congenital relating to a failure of fusion of mesenchymal layers forming the pericardial space. Pericardial cysts are considered rare incidental findings, they are mostly asymptomatic and benign, however life-threatening complications may occur. Here we present a case of a silent pericardial cyst that was discovered by chance while performing transthoracic echocardiography (TTE) for a man who was admitted for myocardial infarction.

## Introduction

Pericardial cysts are uncommon mediastinal masses with a reported incidence of 1 in 100,000 patients [1]. They are known by a variety of names in the medical literature, such as pleural cyst, pericardial cyst, pericardial coelomic cyst, springwater cyst, mesothelial cyst, and thin-walled cyst [2]. Pericardial cysts are most commonly congenital, but other causes such as rheumatic pericarditis, tuberculosis, echinococcosis, traumatic, post-cardiac surgery have been described in the literature. They are frequently discovered incidentally during routine chest imaging. Computerized tomography scan (CT-scan) is the preferred diagnostic modality for diagnosing and evaluating pericardial cysts. The treatment can be conservative especially for small asymptomatic cysts or surgical as for life-threatening cases.

## Patient and observation

**Patient information:** a 68-year-old man, chronic smoker, with no previous cardio-vascular diseases, presented to the emergency department complaining of chest pain for the previous two days.

**Clinical findings:** on examination, the patient was stable with a blood pressure of 120/70 mmHg, a pulse rate of 79 beats per minute, a respiratory rate of 16 beats per minute, and an oxygen saturation of 96% on room air. On chest auscultation, the heart sounds were regular, with no murmurs or pericardial rub, and the lung sounds were normal with no crackles.

**Figure 1:**
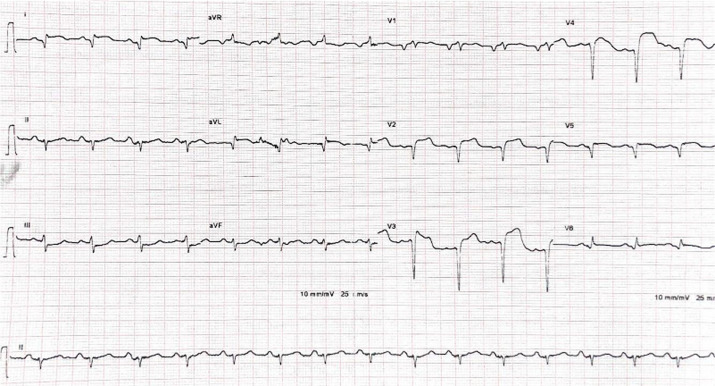
electrocardiogram (EKG) showing ST segment elevation with Q waves in the anterior leads

**Figure 2:**
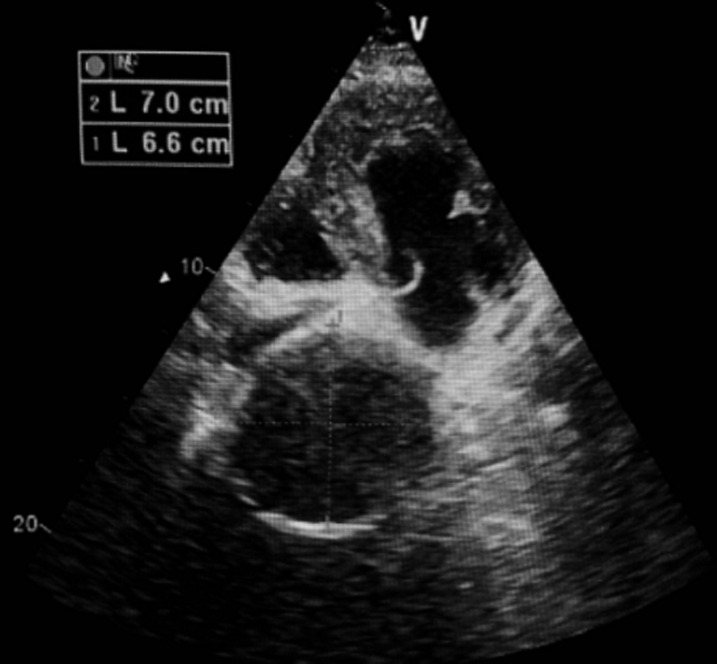
echocardiography: modified apical view showing an echolucent well defined space (70 x 66 mm) next both right and left atrium

**Figure 3:**
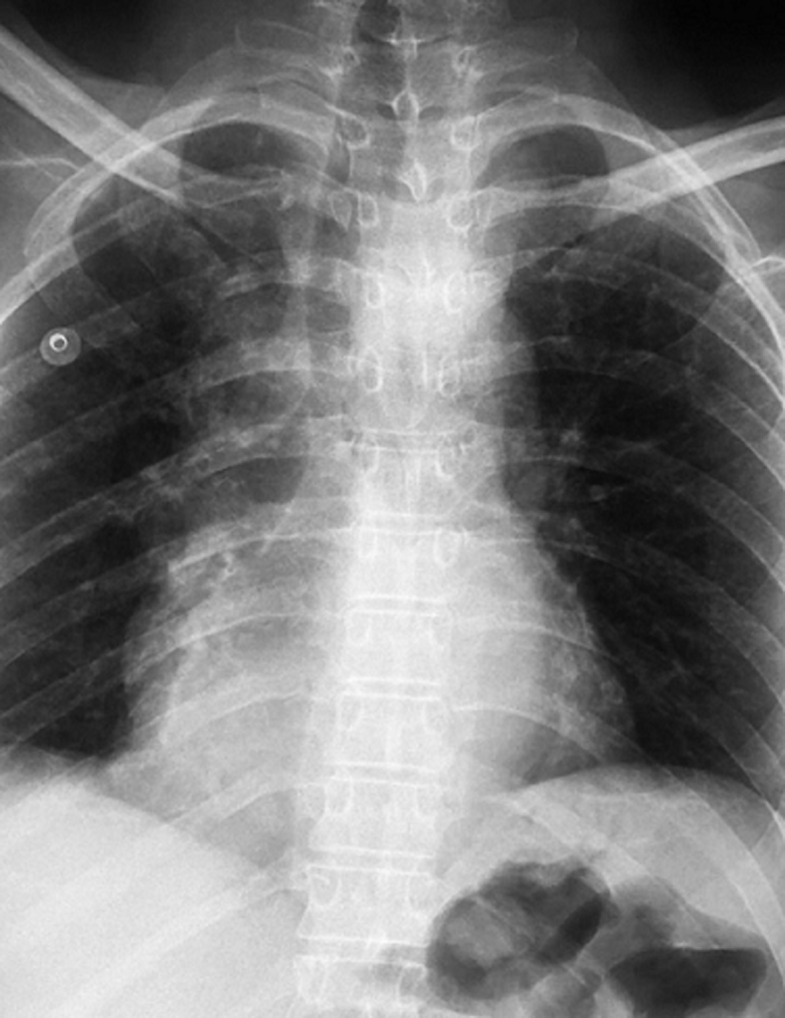
chest X-ray showing homogenous density in the right cardiophrenic angle and trachea deviation

**Figure 4:**
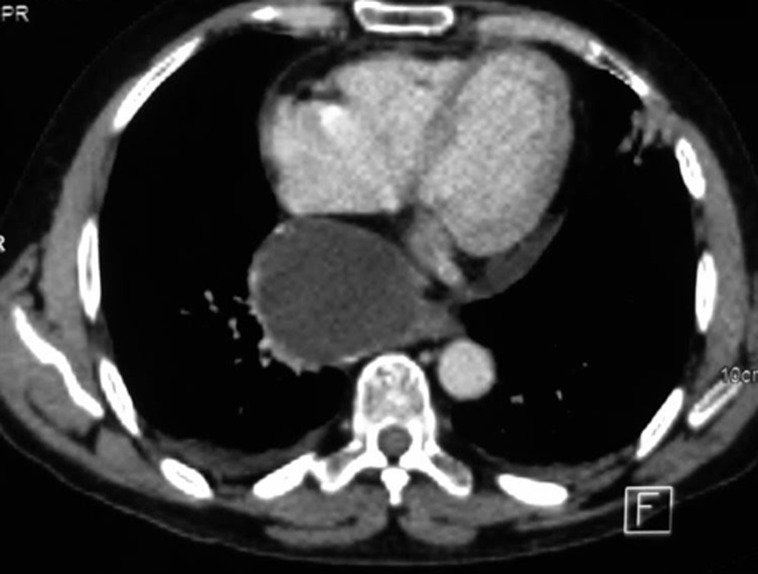
chest CT-scan showing pleuropericardial cyst with 79 x 66 mm at diameter

**Figure 5:**
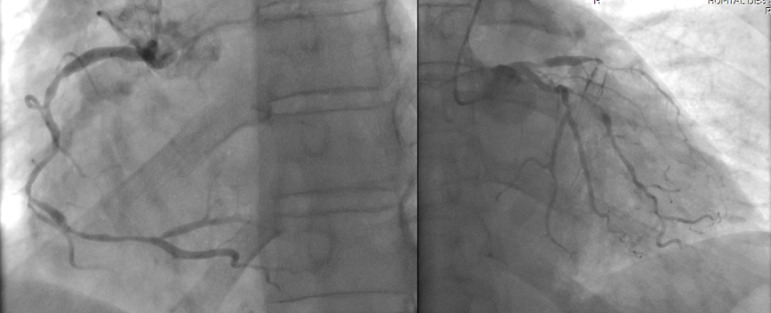
coronarography angiography showing multiple severe stenosis in the left and right coronary arteries

**Diagnostic assessment:** the electrocardiogram revealed ST segment elevation with Q waves in the anterior leads (Figure 1). Transthoracic echocardiography revealed apex, anterior, and anterolateral wall akinesia, with a left ventricular ejection fraction of 37% (Figure 2), modified apical 4-chamber view unexpectedly revealed a well-circumscribed and homogeneous echolucent mass with a diameter of 70 x 66 mm next to both right and left atriums, which lacked color by Doppler. Chest X-ray revealed a homogenous density in the right cardiophrenic angle and trachea deviation (Figure 3) and chest computed tomography confirmed the presence of a pleuropericardial cyst with a diameter of 79 x 66 mm in the right cardiophrenic angle adjacent to both atriums (Figure 4). As the electrocardiogram (EKG) revealed that the patient had a myocardial infarction, coronary angiography was performed, which revealed tri-troncular coronary lesions (Figure 5).

**Diagnosis:** the diagnosis of pleuropericardial cyst was made based on the CT-scan findings. Given the patient's geographical origin and the fact that hydatidosis is still prevalent in Morocco, a hydatid cyst was considered, but hydatid serology was positive.

**Therapeutic interventions and follow up:** surgical treatment consisting coronary bypass and removal of the cyst was explained to the patient however he refused to undergo any surgery. One month after his discharge, the patient was evaluated; he was doing well, with no recurrence of chest pain and no signs of cardiac failure. Transthoracic echocardiography revealed a stationary left ventricular ejection fraction and the same cyst dimensions.

**Informed consent:** the patient gave his verbal informed consent for this case report to be published.

## Discussion

Pericardial cysts are usually asymptomatic and appear as an incidental finding during routine chest X-rays. However, when they compress adjacent organs, symptoms such as chronic cough, chest discomfort, palpitations or dyspnea may occur. Pericardial cysts can lead to serious complications such as cyst rupture, cardiac tamponade [3], heart failure, arrhythmia, torsion and even sudden death [4]. The CT-scan is widely accepted as the primary modality for confirming the diagnosis because it provides excellent delineation of the pericardial anatomy as well as precise localization, size and relationship with nearby structures. Magnetic resonance imaging is another diagnosis modality that can be helpful when a CT-scan is uncertain [5]. When conventional imaging findings are inconclusive, diffusion weighted magnetic resonance imaging may be useful in differentiating symptomatic pericardial cysts from neoplastic and infectious mediastinal lesions [6]. Transthoracic echocardiography is recognized as a follow-up diagnostic modality and image-guided percutaneous aspiration, however TTE is not preferred as the primary diagnostic modality due to the narrow window for visualization and the possibility of missing unusual cyst locations [5].

The treatment of a pericardial cyst is determined by the size, the shape and the occurrence of symptoms, taking into account the patient´s willingness to undergo treatment. Since the vast majority of patients are asymptomatic, treatment is usually conservative with a close follow-up. Video assisted thoracotomy or surgical resection are recommended for patients who are symptomatic, have large cysts, are at risk of complications, or are in a life-threatening emergency [7]. Percutaneous aspiration and ethanol sclerosis are recommended as initial management for congenital and inflammatory cysts [8], they can be used in large cyst waiting surgery to relieve symptoms and for cytological or microbiological evaluation.

In our case, the pericardial cyst was asymptomatic and was discovered by chance while performing TTE for myocardial infraction seen 48 hours after symptom onset. Given this fact, conservative management seems to be the appropriate management, however the coronary angiography revealed tri-troncular stenosis, therefore we decided on surgical treatment that included a coronary bypass and cyst removal, yet the patient refused all surgery, preferring medical treatment and close monitoring.

## Conclusion

Pericardial cysts are a rare entity of mediastinum masses, they are usually asymptomatic and incidentally discovered, therefore conservatively management is mostly recommended, however the occurrence of symptoms or life -threatening complications apply surgical treatment for those cysts.
